# Selected Pathway Analyses to Gain Mechanistic Insights into the Pathogenesis of Feline Hypertrophic Cardiomyopathy

**DOI:** 10.3390/ijms26136497

**Published:** 2025-07-05

**Authors:** Lea Schurna, Jessica Joshua, Josep Monné Rodríguez, Francesco Prisco, Marco Baron Toaldo, Simon De Neck, Francesca Baggio, Sonja Fonfara, Anja Kipar

**Affiliations:** 1 Institute of Veterinary Pathology, Vetsuisse Faculty, University of Zurich, 8057 Zurich, Switzerland; lea.schurna@uzh.ch (L.S.); josep.monne@roche.com (J.M.R.); francesco.prisco@uzh.ch (F.P.); francesca.baggio@uzh.ch (F.B.); 2 Center for Clinical Studies, Vetsuisse Faculty, University of Zurich, 8057 Zurich, Switzerland; 3 Department of Clinical Studies, Ontario Veterinary College, University of Guelph, Guelph, ON N1G 2W1, Canada; jjoshua@uoguelph.ca; 4 Unit of Pathology, Department of Veterinary Medicine and Animal Production, University of Naples Federico II, 80137 Naples, Italy; 5 Clinic for Small Animal Internal Medicine, Division of Cardiology, Vetsuisse Faculty, University of Zurich, 8057 Zurich, Switzerland; marco.barontoaldo@uzh.ch

**Keywords:** cats, hearts, sex, age, myocardium, HCM

## Abstract

Hypertrophic cardiomyopathy (HCM) is the most prevalent acquired heart disease in cats and shares many clinical, phenotypical and pathological features with human HCM. Despite its relevance, knowledge on the pathomechanisms underlying the disease is limited. The present study aimed to characterize the molecular phenotypic changes in cardiomyocytes in feline HCM (fHCM) to better understand their contribution to the pathogenesis. To achieve this, the myocardium of the left ventricular free wall of 15 cats with confirmed fHCM and 30 control cats (two age groups: 16 cats 18-month-old, and 14 older adult cats without cardiac disease) were subjected to RT-qPCRs for markers representative of cardiomyocyte function. Overall, all markers were expressed at the highest level in young control cats, and increasing age correlated with decreased expression, regardless of sex. The comparison between the older adult control cats and those with HCM showed increased transcription levels for most markers associated with the disease, and higher expression of all markers in affected male cats compared to females. The constitutive transcription of all markers provides evidence of continuous myocardial adaptation throughout cats’ life. The high transcription values in the myocardium of young healthy cats and male cats affected by HCM suggest a particularly high myocardial responsiveness early in life and with HCM and reveal sex as relevant factor in the disease process. These results support the relevance of age and sex in the cardiac response to HCM in feline hearts.

## 1. Introduction

Hypertrophic cardiomyopathy (HCM) is the most common cardiomyopathy in cats, with an overall prevalence of approximately 15% which increases to 29% in animals older than 9 years [1,2,3]. The average age of cats at diagnosis is 6 years [2,4,5,6], and male neutered cats carry the highest risk of developing HCM [1,2,4,5,7,8]. HCM is most often diagnosed in Domestic Shorthair cats, but certain breeds are predisposed, including Maine Coon, Norwegian Forest, Sphinx, British Shorthair, and Ragdoll cats [1,7,8,9,10]. In humans, HCM is considered a genetic disease, and numerous HCM-associated mutations have been identified [11]. Genetic mutations linked to HCM have also been reported in cats. To date, three mutations have been identified: the *A31P* and *R820W* mutations in the *MYBPC3* gene in Maine Coon and Ragdoll cats, respectively, and a mutation in the ALMS1 gene in Sphynx cats. Additionally, a mutation in the feline orthologue of the human MYH7 gene has been reported in a Domestic Shorthair cat and is considered a possible cause of HCM in that particular case [12,13,14].

The clinical presentation of feline HCM (fHCM) is heterogeneous [2,7,15,16]; hence, the diagnosis is challenging in many cats, and echocardiography, performed by a veterinary cardiologist, is the clinical diagnostic test of choice [17,18]. Macroscopically, an enlarged heart with marked diffuse or segmental thickening of the left ventricular (LV) free wall, interventricular septum, and/or papillary muscles, a narrowed LV lumen, frequently with dilation of the left atrium and increased heart weight to body weight ratio, are characteristics of fHCM [19,20,21].

Feline HCM is associated with a range of histopathological features in the myocytic, interstitial and vascular compartments that represent remodeling processes [22,23,24]. The myocardial changes are consistent with degeneration, cell loss and repair, ranging from myofiber disarray to replacement fibrosis with associated myofiber loss. This is seen together with a variable degree of interstitial collagen deposition and macrophage accumulation and an overall significant increase in interstitial space [25,26]. In fHCM, the myocardium has a lower capillary density, with often irregularly arranged capillaries, indicating microvascular dysfunction; arteriosclerosis has also been observed [22,25,27]. Interestingly, cardiomyocyte hypertrophy is not a confirmed feature of fHCM [10,20,23,26]. Indeed, morphometric analyses did not find any direct or indirect evidence of an increase in cardiomyocyte size and/or the contractile mass [26,28].

Previous molecular studies by our group have gathered evidence of the pathomechanisms underlying the development of fHCM. In the first step, we identified age-associated variations in the transcription of inflammatory and immunomodulatory cytokines in the healthy feline myocardium; most exhibited a decrease in expression with increasing age while the transcription of TGF-β increased. This indicates a pro-inflammatory environment in younger animals and the transition to a profibrotic environment with age [29,30,31]. We also found first evidence to explain why HCM is more common in male cats: males show higher constitutive transcription of remodeling enzymes (matrix metalloproteinases (MMP)-2 and -3; tissue inhibitors of metalloproteinases (TIMP)-1, -2 and -3) [30]. In a recent transcriptome study, we found variations in the expression of genes reflecting pathways of particular interest considering our previous research, i.e., genes associated with cardiomyocyte growth, protection and adaptation to hypoxia, microvascular changes, and intercellular signaling; we identified similarities with functional pathways reported to be affected in human HCM [32].

Based on these and our other previous findings, we hypothesized that alterations in cardiomyocyte function, differentiation, adaptation and growth, together with changes in vascularization and intercellular signaling play relevant roles in the pathogenesis of fHCM. We also considered the likely high relevance of age- and sex-associated differences in gene expression in the healthy and fHCM myocardium. Therefore, we undertook an in-depth study into the transcription of selected myocardial genes in the LV free wall of young and older adult cats of both sexes with HCM and without cardiac disease, investigating the following myocardial processes and responses: growth (IGF1R and IRS1), hypertrophy (MRAS, PI3K, B-RAF and MFN2), dedifferentiation (MEF2C, GATA4 and GATA6), vascularization (VEGFA and TSP1) and intercellular signaling (ITGAM and ITGA10).

## 2. Results

### 2.1. Study Population and Histological Examination

Forty-five cats were included in the study, 15 cats with HCM, and 30 cats without cardiac disease (control cats).

The age of the HCM cats ranged from 1 to 15 years (mean age: 8.93 years); eleven were male, and eight of these neutered; four cats were female, and three of these neutered (Supplemental Table S1). The histological examination of these hearts showed features consistent with changes described for fHCM [20,22,23,24,25,26]. The most common findings were myocardial disarray and varying degrees of interstitial edema and fibrosis. Replacement fibrosis, characterized by focal to multifocal loss of cardiomyocytes and their replacement by collagen, fibroblasts and blood vessels, was rarely observed.

The control cats comprised two groups. Sixteen animals (1.5 years of age, each eight female and male) represented the young control cats; these had served as healthy control group in a previous study of our group [32]. Their hearts did not exhibit any gross or histological changes. The second group (older adult control cats) comprised fourteen adult cats (10 male, four female). As these were stray cats, their exact age was unknown. Based on dentition, exterior and development, they were considered adults and of a higher age than the young control cats. All these animals exhibited trauma-related lesions but neither gross nor histological changes in the hearts (Supplemental Table S1).

### 2.2. The Feline Myocardium of Both Sexes Constitutively Transcribes Markers Involved in Growth and Hypertrophy, Fetal Genes, and Markers of Vascularization and Intercellular Signaling, with Higher Expression in Young Cats

Two-step RT-qPCRs were established and applied for a range of markers of interest (Table 1) for which we have found evidence of upregulation in a previous RNA sequencing study [32]; these fall into different functional groups, focusing on myocardial growth, hypertrophy, fetal genes, vascularization and intercellular signaling.

**Figure 1:**
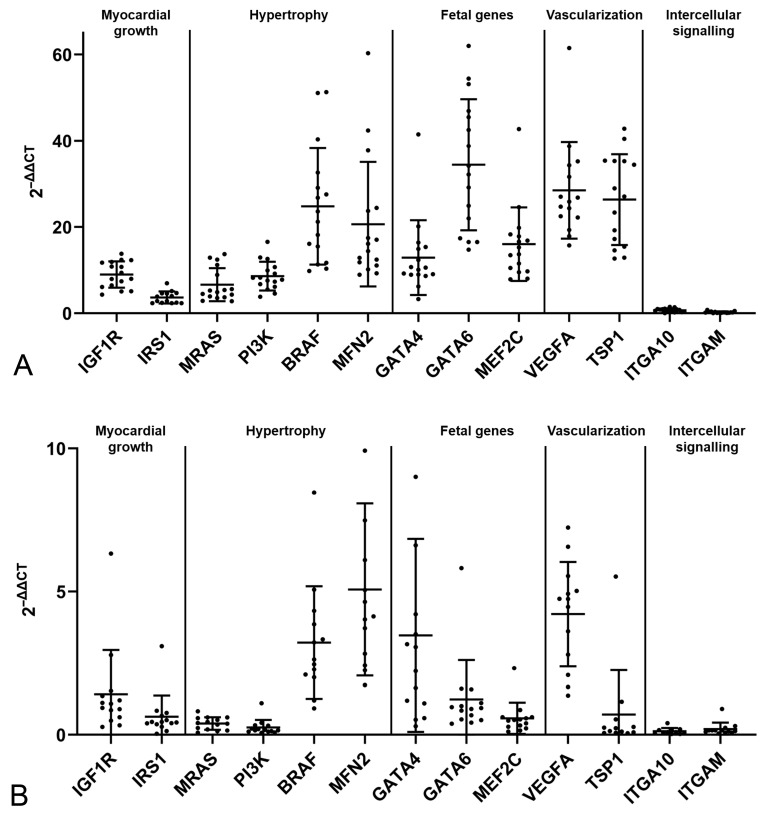
Relative transcription levels of markers for myocardial growth, hypertrophy and fetal genes, and markers of vascularization and intercellular signaling in the myocardium of control cats. (**A**) Values in young control cats (1.5 years; n = 16; each 8 female and 8 male). (**B**) Values in older adult control cats (n = 14; 4 female, 10 male). Dots represent individual animals; RT-qPCRs were performed in duplicates and expression values were normalized against the reference gene GAPDH. ∆∆CT values are calculated by using the 2^−∆∆CT^ method. Data are shown as mean ± standard deviation.

All markers known to be expressed by cardiomyocytes (IGF1R, IRS1, MRAS, PI3K, B-RAF, MFN2, MEF2C, GATA4, GATA6, VEGFA, TSP1 and ITGA10) and the leukocyte surface receptor ITGAM were found to be constitutively transcribed in the healthy myocardium, both in the young and older controls (Figure 1). Among all markers, those involved in cardiomyocyte hypertrophy (B-RAF, MFN2) and the proangiogenic marker VEGFA showed particularly high expression in both control groups; in the young controls, the fetal gene GATA6 and the vascularization marker TSP1 were expressed at high level (Figure 1A), in contrast to GATA4, which showed higher expression in older adult controls (Figure 1B).

Overall, substantial interindividual variability was observed for most markers (Figure 1). For several markers, it was higher in the older adult control cats, which might reflect the greater interindividual difference in this group compared to the young control cats with regard to age and other potentially relevant factors.

**Figure 2:**
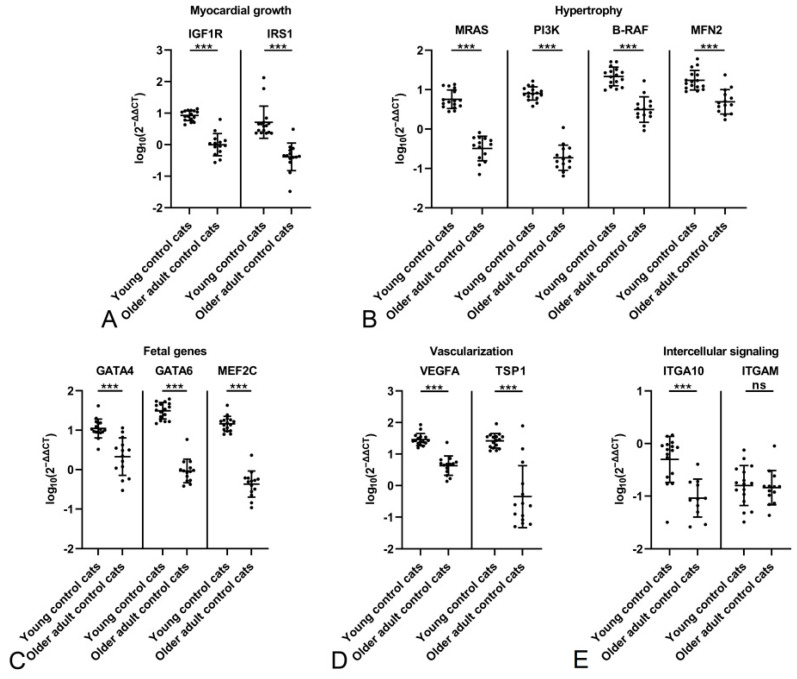
Evaluation of gene expression by qRT-PCR in the myocardium of young control cats (1.5 years; n = 16) and older adult control cats (n = 14). Relative transcription levels of markers for myocardial growth (**A**), hypertrophy (**B**), fetal genes (**C**), vascularization (**D**) and intercellular signaling (**E**). All markers expressed by cardiomyocytes show significantly higher expression levels (all adjusted *p* ≤ 0.001) in the young control cats. For ITGAM, expressed by leukocytes, the difference is not significant (adjusted *p* = 0.964). Dots represent individual animals; RT-qPCRs were performed in duplicates and expression values are normalized against the reference gene GAPDH. ∆∆CT values are calculated by using the 2^−∆∆CT^ method followed by statistical analyses in R including testing for normality (Shapiro–Wilk test), log10 transformation, one-way ANOVA and Tukey post hoc test. Data are shown as mean ± standard deviation. Asterisks indicate the significance level: adjusted *p* ≤ 0.001 (***); ns = non-significant. Detailed information on the statistical results is provided in Table S2.

The myocardium of the young control cats showed significantly higher expression levels (all *p* ≤ 0.001) for all markers expressed by cardiomyocytes, covering various relevant aspects, i.e., myocardial growth (IGF1R, IRS1; Figure 2A), hypertrophy (MRAS, PI3K, B-RAF, MFN2; Figure 2B) and fetal genes (GATA4, GATA6, MEF2C; Figure 2C), vascularization (VEGFA, TSP1; Figure 2D) and intercellular signaling (ITGA10; Figure 2E). For ITGAM, the only marker not expressed by cardiomyocytes but instead by leukocytes, the difference was not significant (*p* = 0.964; Figure 2E), suggesting a similar amount of leukocytes in the unaltered myocardium regardless of age.

**Figure 3:**
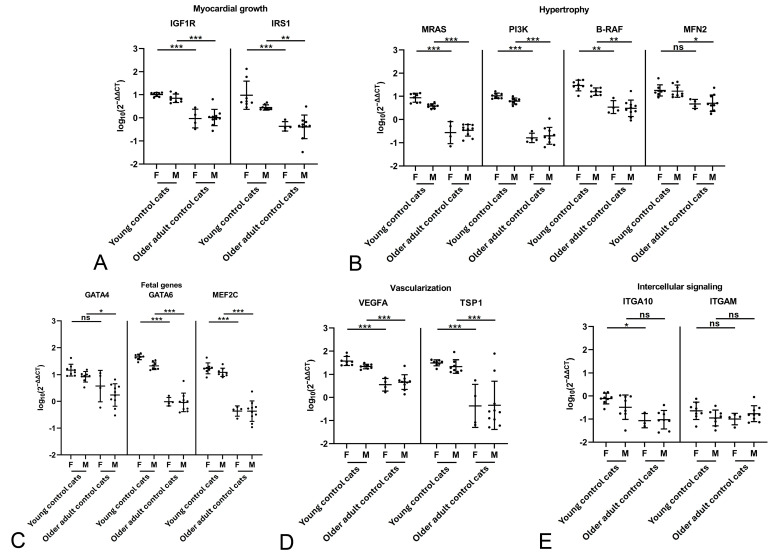
Evaluation of gene expression by qRT-PCR in the myocardium of female (F) young control cats (n = 8) and male (M) young control cats (n = 8) as well as female (F) older adult control cats (n = 4) and male (M) older adult control cats (n = 10). Relative transcription levels of markers for myocardial growth (**A**), hypertrophy (**B**), fetal genes (**C**), vascularization (**D**) and intercellular signaling (**E**). Within both groups, there were no significant differences in the transcription level of any marker between male and female cats (not shown in graph). Almost all markers, except for MFN2, GATA4, and the intercellular signaling factors ITGAM and ITGA10, show significantly higher transcription levels in both female and male young control cats. Dots represent individual animals; RT-qPCRs were performed in duplicates and expression values are normalized against the reference gene GAPDH. ∆∆CT values are calculated by using the 2^−∆∆CT^ method followed by statistical analyses in R including testing for normality (Shapiro–Wilk test), log10 transformation, two-way ANOVA and Tukey post hoc test. Data are shown as mean ± standard deviation. Asterisks indicate the significance level: adjusted *p* ≤ 0.05 (*), adjusted *p* ≤ 0.01 (**) and adjusted *p* ≤ 0.001 (***); ns = non-significant. Detailed information on the statistical results is provided in Table S2.

For both control groups, the potential influence of sex was examined. Within either group, transcription levels of the markers did not differ significantly between male and female cats (Figure 3). A comparison of male and female cats between the two control groups confirmed significantly higher transcription levels for all markers but MFN2, GATA4 and ITGAM in the young female controls, and for all markers but ITGA10 and ITGAM in the young male control cats (Figure 3). This confirms an influence of age rather than sex on constitutive marker expression.

### 2.3. The HCM Myocardium Is Markedly More Dynamic than the Myocardium of Healthy Adult Cats

**Figure 4:**
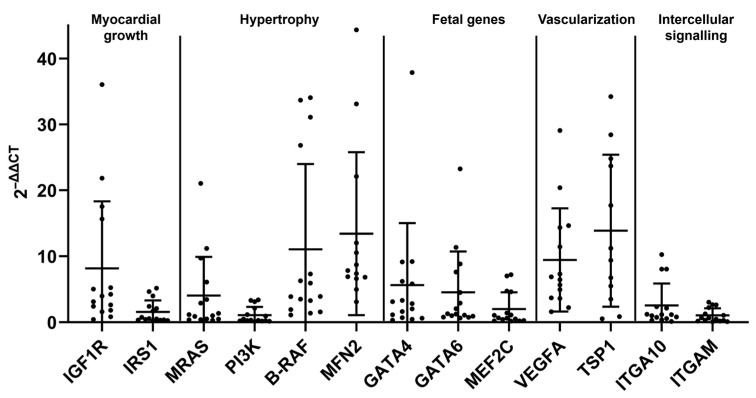
Relative transcription levels of markers for myocardial growth, hypertrophy, fetal genes, vascularization and intercellular signaling in the myocardium of cats with HCM. All markers are transcribed. Dots represent individual animals; RT-qPCRs were performed in duplicates and expression values are normalized against the reference gene GAPDH. ∆∆CT values are calculated by using the 2^−∆∆CT^ method. Data are shown as mean ± standard deviation.

Similar to the control cats, all markers were transcribed in the LV myocardium of HCM cats (Figure 4). As in both control groups, markers of hypertrophy (B-RAF, MFN2) and the angiogenic factor VEGFA showed the highest relative expression. However, instead of GATA4, a fetal gene that was also quite strongly expressed in the older control cats, TSP1, a matrix protein that stimulates angiogenesis, deposition of new matrix and recruitment of inflammatory cells [64,65,66] showed a notably high transcription level, suggesting a role in HCM.

**Figure 5:**
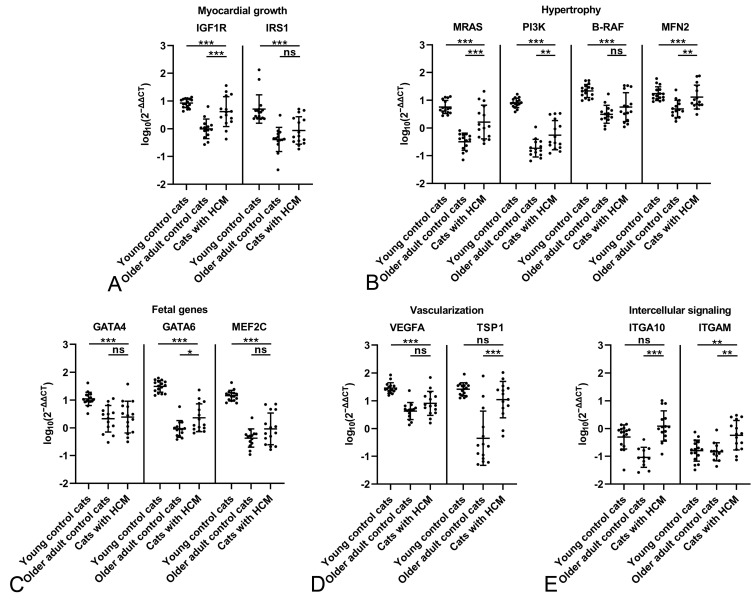
Evaluation of gene expression by qRT-PCR in the myocardium of young control cats (1.5 years; n = 16) and older adult control cats (n = 14) in comparison to cats with HCM (Group 3, n = 15). Relative transcription levels of markers for myocardial growth (**A**), myocardial hypertrophy (**B**), myocardial fetal genes (**C**), vascularization (**D**) and intercellular signaling (**E**). Dots represent individual animals; RT-qPCRs were performed in duplicates and expression values are normalized against the reference gene GAPDH. ∆∆CT values are calculated by using the 2^−∆∆CT^ method followed by statistical analyses in R including testing for normality (Shapiro–Wilk test), log10 transformation, one-way ANOVA and Tukey post hoc test. Data are shown as mean ± standard deviation. Asterisks indicate the significance level: adjusted *p* ≤ 0.05 (*), adjusted *p* ≤ 0.01 (**) and adjusted *p* ≤ 0.001 (***); ns = non-significant. Detailed information on the statistical results is provided in Table S2.

The comparison of cats with HCM and the young control cats showed that the transcription levels in the HCM myocardium generally did not reach those of the young control animals. Indeed, they were significantly lower for all markers of myocardial growth, hypertrophy and fetal genes, and for the vascularization marker VEGFA (Figure 5A–D). Interestingly though, the expression of both integrins (ITGA10, ITGAM) was higher in cats with HCM (Figure 5E), and significantly for ITGAM, consistent with the increased number of leukocytes and particularly macrophages in the myocardium in HCM [25,26].

The comparison between older adult control cats and cats with HCM yielded different results. Most investigated markers were expressed at significantly higher levels in HCM. This included markers for cell growth (IGF1R), hypertrophy (MRAS, PI3K, MFN2), dedifferentiation (GATA6), and vascularization (TSP1) (Figure 5A–D), indicating attempts at maintaining myocardial function. Both intercellular signaling markers ITGA10 and ITGAM were also significantly upregulated (Figure 5E), reflecting the leukocyte accumulation and indicating a more intense cardiomyocyte crosstalk in HCM.

### 2.4. Male Cats with HCM Exhibit a More Dynamic Myocardium than Female Cats with HCM

**Figure 6:**
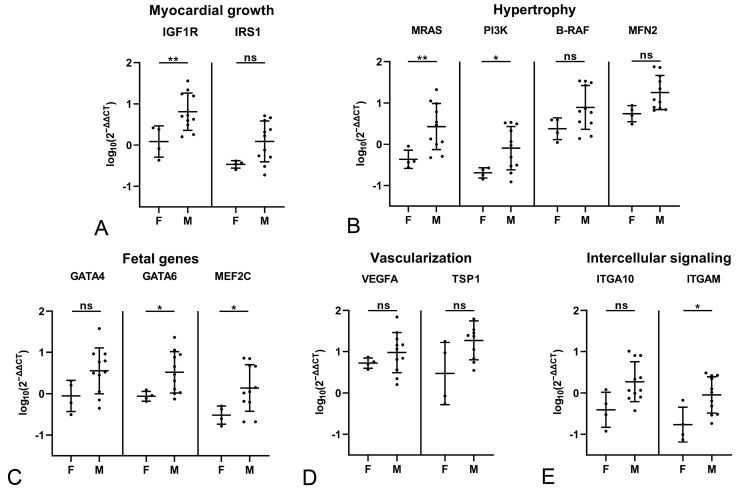
Evaluation of gene expression by qRT-PCR in the myocardium of female (F; n = 4) and male (M; n = 11) cats with HCM. Relative transcription levels of markers for myocardial growth (**A**), myocardial hypertrophy (**B**), myocardial fetal genes (**C**), vascularization (**D**), and intercellular signaling (**E**) in the myocardium of female (F; n = 4) and male (M; n = 11) cats with HCM. All markers show higher transcription levels in male cats with HCM; for some the difference is significant. Dots represent individual animals; RT-qPCRs were performed in duplicates and expression values are normalized against the reference gene GAPDH. ∆∆CT values are calculated by using the 2^−∆∆CT^ method followed by statistical analyses in R including testing for normality (Shapiro–Wilk test), log10 transformation, two-way ANOVA and Tukey post hoc test. Data are shown as mean ± standard deviation. Asterisks indicate the significance level: adjusted *p* ≤ 0.05 (*), adjusted *p* ≤ 0.01 (**); ns = non-significant. Detailed information on the statistical results is provided in Table S2.

Although the factor “sex” was not associated with statistically significant differences in marker expression in the control groups, it was investigated in HCM, as the disease is more prevalent in male cats [1]. This indeed revealed a significant sex-specific difference within each marker group for cats with HCM. Male HCM cats showed significantly higher transcription levels for myocardial growth (IGF1R) and hypertrophy markers (MRAS, PI3K), fetal genes (GATA6, MEF2C) as well as the intercellular signaling gene ITGAM (Figure 6). Also, while the difference was not significant, there was a tendency towards higher expression of all other markers (IRS1, B-RAF, MFN2, GATA4, VEGFA, TSP1 and ITGA10) in males (Figure 6).

**Figure 7:**
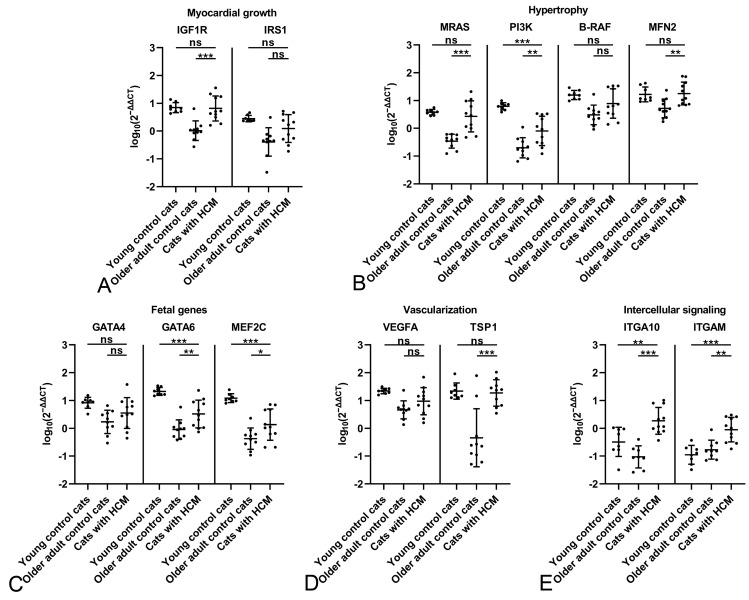
Relative transcription levels of markers for myocardial growth (**A**), myocardial hypertrophy (**B**), myocardial fetal genes (**C**), vascularization (**D**), and intercellular signaling (**E**) in the myocardium of male young control cats (n = 8) and older adult control cats (n = 10) and in male cats with HCM (n = 11). Dots represent individual animals; RT-qPCRs were performed in duplicates and expression values are normalized against the reference gene GAPDH. ∆∆CT values are calculated by using the 2^−∆∆CT^ method followed by statistical analyses in R including testing for normality (Shapiro–Wilk test), log10 transformation, two-way ANOVA and Tukey post hoc test. Data are shown as mean ± standard deviation. Asterisks indicate the significance level: adjusted *p* ≤ 0.05 (*), adjusted *p* ≤ 0.01 (**) and adjusted *p* ≤ 0.001 (***); ns = non-significant. Detailed information on the statistical results is provided in Table S2.

When comparing transcription levels in male control cats of both age groups with male HCM cats, the upregulation of all markers in HCM versus older adult controls (Figure 5) was confirmed; the difference was also significant for the fetal gene marker MEF2C (Figure 7).

**Figure 8:**
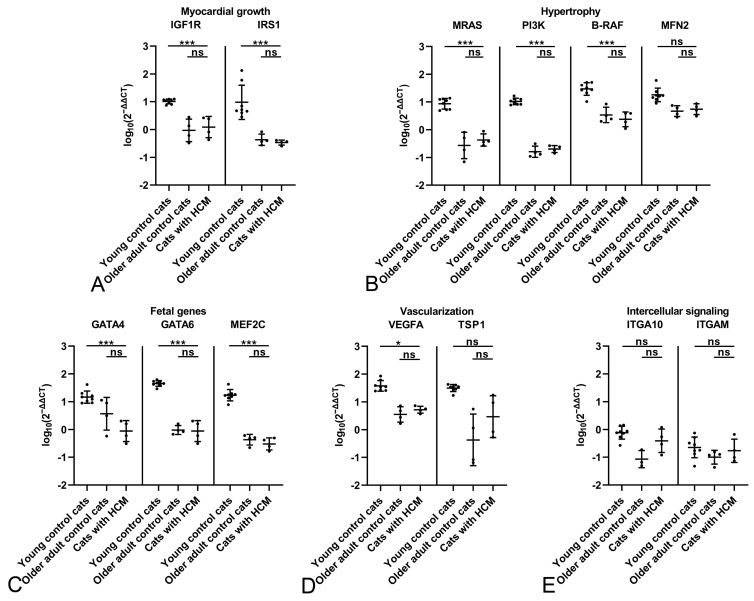
Relative transcription levels of markers for myocardial growth (**A**), myocardial hypertrophy (**B**), myocardial fetal genes (**C**), vascularization (**D**), and intercellular signaling (**E**) in the myocardium of female young control cats (n = 8) and older adult control cats (n = 4) and female cats with HCM (n = 4). All markers show no difference in the relative expression levels when comparing the older adult control animals and those with HCM. Dots represent individual animals; RT-qPCRs were performed in duplicates and expression values are normalized against the reference gene GAPDH. ∆∆CT values are calculated by using the 2^−∆∆CT^ method followed by statistical analyses in R including testing for normality (Shapiro–Wilk test), log10 transformation, two-way ANOVA and Tukey post hoc test. Data are shown as mean ± standard deviation. Asterisks indicate the significance level: adjusted *p* ≤ 0.05 (*) and adjusted *p* ≤ 0.001 (***); ns = non-significant. Detailed information on the statistical results is provided in Table S2.

For female cats, no significant differences were found when comparing the older adult control animals and those with HCM (Figure 8). However, the low number of cats in both groups (each n = 4) needs to be considered and therefore the results must be interpreted with caution. The highest overall transcription values were found in the young control cats, the same as for the male young control cats (Figure 7 and Figure 8).

## 3. Discussion

The primary aim of this study was to expand the understanding of the pathogenesis of fHCM, the most common acquired heart disease in cats [17]. This was attempted by examining the transcriptional profile of selected pathways, with a focus on myocardial growth, hypertrophy and fetal gene expression, vascularization and intercellular signaling, in cats with HCM and non-affected maturing and full-grown cats. The investigation of the unaltered myocardium served to gather information on the constitutive transcription profile and to assess dynamic changes in the myocardium over the cat’s adult life, while also acknowledging the fact that age is a known associated factor in fHCM [7,15,16]. Furthermore, including both sexes allowed an investigation of the potential impact of sex, since HCM is known to mainly be a disease of older male cats [72].

We generally found the highest relative expression levels for all investigated cardiomyocyte-related markers in the young, 1.5-year-old control animals. This is consistent with previous findings of age-associated gene expression in cats without heart disease, in which myocardial cytokine, MMP and TIMP mRNA concentrations were found to be negatively correlated with age [30]. The findings indicate that the feline myocardium is still developing, and possibly maturing, at the age of 18 months, allowing further cardiomyocyte growth and differentiation. The particularly high expression of VEGFA, GATA6 and TSP1 also indicates that myocardial vascularization is still in process at this age [62,63,66]. Interestingly, intercellular signaling markers (ITGAM and ITGA10) were expressed at relatively low levels. In conclusion, in maturing healthy cats (represented here by a group of 18-month-old animals), the myocardial environment appears to focus on the adaptation of cardiomyocytes to the adult stage, possibly to achieve optimal cardiovascular function. In the older adult, hence overall more mature control group, constitutive transcription was still observed, though generally at a significantly lower level than in the younger cats. This indicates a less dynamic myocardium with age, as part of a potential general senescence process [73,74]. However, GATA4 transcription seemed to be of relevance in this group. Considering that GATA4 regulates the expression of genes involved in myocardial contraction and promotes cardiac hypertrophy in response to hemodynamic stress [58], this could be interpreted as a sign of myocardial maintenance or the readiness to adapt to changing hemodynamics.

The older adult control group showed higher interindividual variability in the transcription of several markers. This might reflect the greater interindividual differences in terms of age and environmental factors, as these cats did not live in a controlled environment like the young control cats. On the other hand, the increased variance might also reflect a variable transcriptional responsiveness to pathological conditions, i.e., possible subclinical systemic diseases that could have influenced the myocardial gene expression [30]. We also examined the control hearts for differences in transcription levels between male and female cats. This revealed no significant differences between both sexes which appears to differ from the cellular and microenvironmental sex differences observed for human hearts [75]. Since we could examine an equal number of intact cats of both sexes in the 18-month-old control group (both n = 8), these results can be considered as reliable. For the more variable older control group, the results are less meaningful, as only four female cats were available for the comparison with ten male cats.

Overall, the myocardium of cats with HCM showed higher transcription levels for all markers than that of the older adult control cat group. The comparison between older controls and cats with HCM is of particular interest, as these groups are overall of comparable maturity. It revealed significant upregulation of markers in all functional categories with HCM. This was most pronounced for IGFR1, MRAS, TSP1, ITGA10 and ITGAM. In fact, the transcription levels of the two intercellular signaling markers ITGA10 and ITGAM were equal to and higher, respectively, than those in the young controls which otherwise showed higher levels for all markers. This finding is not only in line with our observation of a leukocyte accumulation in the HCM myocardium [26] but also indicates more intense cardiomyocyte crosstalk in HCM.

Besides the intercellular signaling markers, the transcription of IGFR1, MRAS and TSP1 was most intensely upregulated with HCM. Considering the degenerative and reparative processes that affect the myocardium in HCM (i.e., myocardial disarray, cardiomyocyte loss, replacement fibrosis), the upregulation of IGF1R and MRAS, both activators of the PI3K and MAPK pathway [33,41], might represent a compensatory reaction of the remaining cardiomyocytes in the attempt to maintain cardiac function. In aged mice, IGF1R activation resulted in an increased maximum lifespan, as it correlated with suppressed autophagic flux and impaired oxidative phosphorylation in the heart [76].

The upregulation of TSP1 indicates involvement of the extracellular matrix (ECM), as TSP1 interacts with MMPs and TIMPs, main players in myocardial matrix remodeling [31], and is an activator of TGF-β [77]. As a cytokine, TGF-β plays a crucial role in the regulation of various processes, including fibroblast proliferation and increased collagen synthesis/deposition in the ECM, as well as promotion and inhibition of angiogenesis and endothelial cell proliferation depending on the respective microenvironment [77,78].

This links to previous findings in fHCM, i.e., interstitial collagen deposition and replacement fibrosis [22,25,26] and is in line with the equally elevated expression of ITGA10 and its role in cell adhesion and ECM interactions to maintain the structural integrity of the myocardium [70]. Since recruitment of inflammatory cells is described as another key role of TSP1 and macrophages are its main source, at least in the context of wound healing, the elevated TSP1 mRNA expression is likely linked to the interstitial accumulation of macrophages in cats and acts in synergy with the macrophage recruitment by ITGAM [26,64,68].

Given that GATA6 transcription is likewise significantly elevated in cats with HCM, the assumption that both TSP1 and GATA6 contribute not only to the fibrosis but also to the frequently described vascular changes is plausible [61,62,65,66].

Different from the myocardium of the control cats, the myocardium of cats with HCM exhibited clear sex-related differences. Indeed, there was an obvious tendency for higher marker expression (for more than half of the markers with statistical significance) in male cats with HCM in comparison to female cats with HCM. These findings suggest that various adaptive processes for cardiomyocyte preservation, myocardial integrity and maintenance of cardiac function in response to myocardial injury are influenced by sex. Interestingly though, this appears not to be reflected in differences in the gross or histopathological features of HCM in male vs. female cats (personal observation).

Overall, the upregulation of the marker transcription in male but not female HCM hearts compared to the older adult control cat myocardium, indicates sex-dependent differences in cardiac responsiveness during cardiac remodeling processes, potentially influencing progression and presentation of the disease. These differences are in line with previous results that found higher myocardial transcription of pro-inflammatory cytokines and remodeling enzymes in male cats with HCM than in females [26,30,31]. However, so far there is no evidence that the significantly higher intercellular signaling factor ITGAM mRNA levels in the male HCM cats modulate the reported elevated leukocyte and especially macrophage numbers in the HCM myocardium [26]; similarly, it cannot be said that the activation of macrophage pathways observed in the myocardium of male cats with HCM might convey sex-related differences in myocardial remodeling, since affected female cats were not included into these studies [31,79]. Our recent transcriptomics studies highlighted the particular importance of macrophages in the fHCM myocardium; macrophage activation pathways were found to be upregulated, and were highly connected to other pathways suggesting their involvement in fHCM remodeling processes [32]. The results of this and the current study further indicate that HCM in male cats is associated with an increased level of IGF1R and MRAS and thus an enhanced compensatory reaction with activation of the PI3K and MAPK pathway [32].

Interestingly, while both the downstream molecules MEF2C and PI3K showed concordant increased transcription levels, indicating activation of the hypertrophic gene program in cardiomyocytes, and GATA6, a transcription factor regulating the hypertrophic response, was also significantly upregulated, cardiomyocyte hypertrophy is not an overt morphological feature of fHCM [26,28].

The reason for the sex-related differences identified in this study is not known and whether this is associated with a sex specific clinical disease presentation and progression, as reported in humans [75,80], has not yet been studied in cats.

The question whether this observation is the cause or the consequence of the predisposition for male cats to develop HCM cannot be answered at this point. The influence of sex on cardiac remodeling, the relevance of the observed gene transcription pattern in the initiation and progression of HCM in particular in male cats, and the role or cardiomyocytes in disease progression require further research.

## 4. Materials and Methods

### 4.1. Animals

The case material comprised 15 cats with confirmed HCM and 30 control cats. All cats with HCM (cats 1.1 to 1.15) had been presented to the Division of Cardiology, Clinic for Small Animal Internal Medicine, Vetsuisse Faculty, University of Zurich, Switzerland. HCM was clinically diagnosed by a veterinary cardiologist using routine echocardiographic examinations in non-sedated cats [19,81]. Eleven cats (cats 1.1–1.4, 1.9–1.15) presented clinical signs of respiratory distress, and four showed paresis of the hind limbs (cats 1.5–1.8); in one of the latter cats (cat 1.5) a post mortem examination was performed, which confirmed a saddle thrombus in the terminal branch of the abdominal aorta. Cats were euthanized due to poor prognosis. They were either subjected to a full post mortem examination or a heart dissection with owners’ consent, for confirmation of the clinical diagnosis and identification of other disease processes.

The project was subjected to an institutional ethics review and approved by the Ethics Committee of the Faculty of Medicine, University of Zurich (MeF-Ethik-2023-05). Information on individual animals including the main clinical signs is provided in Supplemental Table S1.

Two cohorts of control cases, i.e., without cardiac disease, were included in the study. The first cohort (cats 2.1 to 2.14) were stray cats either found dead or with fatal disease, requiring euthanasia, in the streets of Naples. All cats were subjected to a full diagnostic post mortem examination upon the request of the local authority (Centro di Riferimento regionale per l’Igiene Urbana Veterinaria), to determine the cause of death and the main pathological findings. For these animals, the age was not known, hence they were classified as adults based on the state of the dentition and development (subsequently referred to as “older adult control cats”). Information on individual animals including the main diagnoses is provided in Supplemental Table S1. The second cohort (cats 3.1 to 3.16) comprised Domestic Shorthair cats aged 1.5 years, eight females (cats 3.1–3.8) and eight males (cats 3.9–3.16) (subsequently referred to as “young control cats”). These cats were part of a healthy research population from a commercial laboratory. Their hearts were not required by the company and donated to the University of Guelph. The cats were classified as healthy based on physical examination, complete blood count, routine biochemical tests and post mortem examination performed by a veterinary pathologist or a veterinarian supervised by a veterinary pathologist.

### 4.2. Tissue Sampling

Immediately but generally not later than 2 h after death, the hearts were removed during a heart-only or full post mortem examination. They were grossly examined, then dissected following the route of blood flow or sectioned longitudinally corresponding to the echocardiographic four-chamber view, as described in our previous study [25]. A tissue sample (up to 0.3 cm in diameter) was taken from the myocardium of the LV free wall and immediately immersed in RNAlater (Thermo Fisher Scientific™, Waltham, MA, USA), then stored at −80 °C. The hearts were fixed in 4% neutral buffered formalin for 36 to 72 h, trimmed and routinely embedded in paraffin wax. Sections (2–3 µm) were prepared and routinely stained with hematoxylin and eosin for histological examination.

### 4.3. Real-Time Quantitative Polymerase Chain Reaction (RT-qPCR)

A two-step RT-qPCR protocol was designed and optimized for a range of markers of interest that fall into different functional groups, focusing on myocardial growth, hypertrophy, fetal genes, vascularization and intercellular signaling (Table 1).

#### 4.3.1. Primer and Probe Design

For the different genes, the respective feline genome sequence was selected, and a multiple sequence alignment was performed for genes with transcript variants using Clustal Omega v1.2.4 [82] of the European Molecular Biology Laboratory-European Bioinformatics Institute (EMBL-EBI) to identify any potential differences in the nucleotide base sequence, followed by determining a sequence range for primer design.

Primers and probes were designed using the primer designing tool Primer Blast [83] of the National Center for Biotechnology Information (NCBI). They were designed to span an exon-exon junction, thereby preventing accidental and unintended amplification of genomic DNA (gDNA). The primers and probes generated by Primer Blast were evaluated using the general guidelines for the manual design of primers and probes for quantification assays [84], and the two primer pairs best suited for the corresponding gene were further tested.

For verification of the GC content, optimal primer melting temperature and PCR annealing temperature, the Tm Calculator [85] was applied with the primer concentration of 900 nM later used in the RT-qPCR. To avoid adverse secondary structures, the primers and corresponding probe were analyzed using the Multiple Primer Analyzer [86] and the Oligonucleotide Properties Calculator [87].

When required, the primers were manually optimized with the Primer Express software v3.0.1 [88] by individually adjusting their position by a few bases and then retested for melting and annealing temperature, GC content and unfavorable secondary structures. The primers finally selected were analyzed using BLAST v1.30+ [89] to verify their specificity ensuring that no match with other sequences existed. All designed primers and probes (Table 2) were produced by Microsynth (Balgach, Switzerland). Probes were labeled at the 5′ end with the reporter dye FAM (6-carboxyfluorescein) and at the 3′ end with the quencher dye TAMRA (6-carboxytetramethylrhodamine).

#### 4.3.2. RNA Extraction

For RNA extraction, the RNeasy^®^ Fibrous Tissue Mini Kit (Qiagen, Venlo, The Netherlands) was used according to the manufacturer’s protocol. Disruption and homogenization were performed using the Precellys 24 Tissue Homogenizer (Bertin Technologies SAS, Montigny-le-Bretonneux, France) and 2 mL microtubes containing 1.4 mm ceramic beads (Omni International, Kennesaw, GA, USA) and 300 μL RLT buffer with 1% β-Mercaptoethanol (Sigma-Aldrich, Buchs, Switzerland) while respecting the usage limits of the Precellys^®^ Lysing Kits for liquid homogenization. For centrifugation, 2 mL Eppendorf Safe-Lock^®^ Tubes (Thermo Fisher Scientific™, USA) and 450 μL of highest purity ethanol (Honeywell Riedel-de-Haën™, Fisher Scientific AG, Reinach, Switzerland) were used. The final RNA collection was performed by elution with 40 μL RNase-free water in 2 mL Eppendorf Safe-Lock^®^ Tubes. The quantity of the extracted RNA was measured with the Qubit 4 Fluorometer (Thermo Fisher Scientific™, USA) using the RNA High Sensitivity (HS) Assay Kit (20–100 ng range) following the General Qubit Assay Protocol (Thermo Fisher Scientific™, USA).

#### 4.3.3. DNase Treatment

An additional DNase treatment to remove gDNA contamination was applied to the extracted RNA samples by using the ezDNaseTM enzyme and following the manufacturer’s instructions (Thermo Fisher Scientific™, USA) using the Biometra TRIO Combi Thermocycler (Biometra GmbH, Göttingen, Germany).

#### 4.3.4. cDNA Synthesis

For the generation of single-stranded cDNA, the High-Capacitiy cDNA Reverse Transcription Kit (Thermo Scientific™, USA) was used following the manufacturer’s protocol. All reverse transcription (RT) runs included a minus RT-control to enable the detection of DNA contamination. The master mix of the RT-control contained all reaction components except the RT enzyme mix with the reverse transcriptase, thereby excluding RT to take place. The reaction volume included 14.2 µL of the DNase treated RNA samples. When necessary, the RNA was diluted with water of molecular biology grade (AppliChem GmbH, Darmstadt, Germany) to achieve a maximum concentration of 2 µg per 20 µL. Samples were run on a Biometra TRIO Combi Thermocycler (Analytik Jena GmbH+Co, Jena, Germany) following the recommended thermal cycler conditions. The final cDNA was diluted with water of molecular biology grade to achieve a concentration of 15 ng/µL (calculation based on the RNA quantities from the Qubit measurement) and was immediately subjected to the polymerase chain reaction (PCR) or was short time stored at −20 °C.

#### 4.3.5. Conventional Polymerase Chain Reaction

Each primer pair was tested in a conventional PCR on a thermal gradient to optimize for the annealing temperature. The master mix consisted of 10 μL TaqMan™ Fast Universal PCR Master Mix (2×), no AmpErase™ UNG (Thermo Fisher Scientific, USA), 0.9 μL per primer (10 μM), and 6.2 μL water of molecular biology grade, resulting in a final volume of 20 μL after addition of 2 μL sample cDNA (30 ng of DNA). Samples were run on a Biometra TRIO Combi Thermocycler under the following thermal profile: (1) initial denaturation at 95 °C for 20 s; (2) denaturation at 95 °C for 3 s; and (3) annealing at either 60 °C, 61.8 °C, 63 °C or 64 °C, for 30 s based on the optimal PCR annealing temperatures determined during the primer design. Steps two to three were repeated 45 times. All PCR protocols were applied in parallel to cDNA from LV samples of two cats not included in the present study with two sets of samples each, one containing the reverse transcriptase (RT+ reaction) and one without the enzyme (RT− reaction).

#### 4.3.6. Gel Electrophoresis

Following routine protocols, PCR products combined with DNA Gel Loading Dye (6×) (Thermo Scientific™, USA) were loaded into 1.5% agarose gel (Tris-acetate-EDTA [TAE] buffer, agarose (UltraPureTM Agarose, Invitrogen, Carlsbad, CA, USA, 1× GelRed nucleic acid gel stain (Biotium, Fremont, CA, USA)) and subjected to gel electrophoresis, using GeneRuler 100 bp Plus DNA Ladder (Thermo Fisher Scientific™, USA). Distinct bands matching the molecular weight of the expected PCR product that were detected using the BioDoc-ItTM Imaging System (Fisher Scientific AG, Switzerland) were extracted from the gel, and the obtained DNA product was purified with the GeneJET Gel Extraction Kit (Thermo Fisher Scientific™, USA) according to the provided purification protocol. The resulting DNA was measured with the Qubit using the High Sensitivity (HS) assay for dsDNA and the General Qubit Assay Protocol recommended for the obtained sample concentration. In order to confirm for amplification of the correct target sequences, the purified amplification product was subjected to Sanger sequencing Microsynth (Balgach, Switzerland). Samples that could not be sequenced were subjected to a second run of PCR and gel electrophoresis under the same conditions. The PCR product underwent purification using the GeneJET PCR Purification Kit (Thermo Fisher Scientific™, USA) according to the purification protocol (Protocol A) and was forwarded to Microsynth for sequencing. All obtained sequences were analyzed using NCBI BLAST to confirm the expected sequences.

#### 4.3.7. Quantitative Polymerase Chain Reaction

According to the results of PCR and gel electrophoresis, RT-qPCRs were performed with the annealing temperature showing the highest fluorescence (60 °C). Primers and probes were tested in six dilutions of a 5-fold dilution series to check their efficiency and reproducibility. For dilutions 1:5 to 1:5^2^ samples were run in duplicates and for dilutions 1:5^3^ to 1:5^6^ in triplicates. Each qRT-PCR consisted of 10 μL TaqMan™ Fast Universal PCR Master Mix (2×), no AmpErase™ UNG (Thermo Fisher Scientific, USA), 0.9 μL per primer (900 μM final concentration μM), 0.5 μL probe (500 μM final concentration), and 5.7 μL water of molecular biology grade and 2 μL sample cDNA (30 ng of DNA) resulting in a final volume of 20 μL. All samples were tested in duplicate in MicroAmp™ Fast Optical 96-well reaction plates (Applied Biosystems, Thermo Fisher Scientific, USA). RT-qPCRs were performed on an Applied Biosystems 7500 Fast Real-Time PCR System (Thermo Fisher Scientific, USA) and visualized by the Applied Biosystems 7500 Software v2.0.6.

The default settings for the thermal cycler protocol were used for all qPCRs: (1) 95 °C for 20 s; (2) 95 °C for 3 s; and (3) 60 °C for 30 s (steps 2 and 3 were repeated 40 times). A specific manually set threshold was applied for repeated runs of the same target. All final protocols had an efficiency of >92% and an R^2^ value of >0.915, with the majority showing an efficiency of >98% and an R^2^ value between 98 and 100%, except for B-RAF, ITGAM, and ITGA10 due to their lower sensitivity (sensitive up to a sample dilution of 1:5^2^).

### 4.4. Statistical Analysis

All data were exported into a Microsoft Excel spreadsheet (Microsoft Excel, 2023 Microsoft Corporation, Redmond, WA, USA). The Applied Biosystems 7500 Software v2.0.6 was used to determine the CT values. Relative mRNA transcription levels were calculated using the 2^−∆∆CT^ method [91]. The average CT of each sample was calculated based on the duplicates and normalized to GAPDH as the internal reference (∆CT). Subsequently, the average ∆CT of the adult cat control group was determined to calculate the ∆∆CT by subtracting the average ∆CT of the controls from each ∆CT of the affected animals. Statistical analyses were performed in R (version 4.3.2) [92], using RStudio (version 2023.09.0) [93] with the following packages: tidyr (version 1.1.4) [94], stringr (version 1.5.1) [95], ggplot2 (version 3.4.4) [96] and dplyr (version 1.1.4) [97]. The statistical analysis included testing for normality of the data (Shapiro–Wilk test) and log10 transformation to reach normal distribution of the data. Briefly, applying the Shapiro–Wilk test on the raw data revealed that several groups were not assuming a normal distribution. The raw data were subjected to log10 transformation and the normality assumption was tested again with the Shapiro–Wilk test which showed that the majority of the groups assumed a normal distribution after transformation. Therefore, the statistical analyses were performed on log10 transformed data with parametric tests. For each marker, the statistical analysis was carried out with both a one-way (group) and a two-way (group*sex) ANOVA, to investigate the effect of the group, and the group and sex, respectively. In each case, this was followed by a Tukey post hoc test. Significance was defined as *p*-value (or adjusted *p*-value) ≤ 0.05. Detailed information on the statistical results is provided in Table S2.

## 5. Conclusions

The feline myocardium constitutively transcribes mediators of cardiomyocyte growth, hypertrophy and dedifferentiation, vascularization and intercellular signaling, indicating continuous myocardial adaptation throughout cats’ life. The high transcription values in the myocardium of young healthy cats and male cats affected by HCM suggest a particularly high myocardial responsiveness early in life and with HCM, also revealing sex as relevant factor in the disease process. These results support the relevance of age and sex in the cardiac response to HCM in feline hearts.

## Figures and Tables

**Table 1:** Target markers investigated in feline hearts by RT-qPCR, with their characteristics and function.

Functional Group	Marker	Characteristics and Function
Myocardial growth	IGF1R	Transmembrane receptor. Activates PI3K pathway and MAPK pathway [33], thereby promoting cell proliferation, differentiation, survival and apoptosis [34,35,36].
	IRS1	Signaling protein. Activated by INSR and IGF1R. In addition to effects of IGF1R, insulin signaling is involved in several cardiac regulatory processes, such as cardiomyocyte growth, contractility, apoptosis and increased myocardial perfusion through vasodilation [37,38,39].
Hypertrophy	MRAS	GTPase. Activates PI3K and the MAPK signaling pathways, thereby regulating a variety of cell functions (proliferation, differentiation, survival and apoptosis), including the activation of transcription factors involved in the hypertrophic gene program, such as MEF2c and GATA4, and leading to fetal cardiac gene expression [40,41,42].
	PI3K	Protein kinase. Intracellular signaling protein involved in the PI3K/AKT/mTOR signaling pathway, thus regulating cell growth, proliferation and metabolism [43,44]; did cause cardiac and myocyte hypertrophy in mice when overexpressed [44,45].
	B-RAF	Serine/threonine-specific protein kinase. Activation of ERK1/2. Facilitates cardiac adaptation to wall stress and promotes compensatory hypertrophy of cardiomyocytes [46,47].
	MFN2	Transmembrane GTPase, located in the outer mitochondrial membrane. Contributes to mitochondrial dynamics (fusion, fission) [48,49,50]. Essential for embryonic and postnatal heart development and maintenance of cardiac function [51,52,53,54].
Fetal genes	MEF2C	Transcription factor. Signaling can result in cardiomyocyte hypertrophy accompanied by sarcomeric derangement and elongation [40,55,56,57].
	GATA4	Transcription factor. Crucial for cardiac morphogenesis by regulating cardiac protein expression [58]. Overexpression preserves cardiac function after cardiac injury [59].
	GATA6	Transcription factor. Expressed in both the developing and adult heart, regulates the same hypertrophic response as GATA4 [58,60], while also playing an important role in migration, differentiation, and modulation of vascular smooth muscle cells (VSMCs) [61,62].
Vascularization	VEGFA	Signaling protein and angiogenic factor. Promotes cardiac stem cell migration via the PI3K/Akt signaling pathway [63].
	TSP1	Matricellular protein. Antagonizes VEGFA and activates latent TGF-β which stimulates extracellular matrix deposition, angiogenesis and recruitment of inflammatory cells [64,65,66]. Increased expression in the myocardium of aging mice suggests contribution to age-related cardiac fibrosis [67].
Intracellular signaling	ITGAM	Cell surface receptor located on leukocytes. Potential contribution to the development of cardiac fibrosis through recruitment of fibroblast-activating macrophages [68,69].
	ITGA10	Cell surface receptor on cardiomyocytes. Binds fibrillar and non-fibrillar collagens. Can trigger force-dependent cellular responses, including cell growth and myofibroblast differentiation. Critical for maintaining cardiomyocyte membrane integrity and subsequent protection against dysfunction, death, and fibrosis [70,71].

Abbreviations: RT-qPCR: Real-time quantitative polymerase chain reaction; IGF1R: Insulin-like growth factor 1 receptor; IRS1: Insulin receptor substrate 1; INSR: Insulin receptor; MRAS: RAS-related protein M-Ras isoform; PI3K: Phosphatidylinositol 3-kinase; B-RAF: Serine-threonine-protein kinase B-raf; ERK1/2: Extracellular signaling-regulated kinase 1/2; MAPK: mitogen-activated protein kinase; MFN2: Mitofusin 2; MEF2C: Myocyte enhancer factor 2C; GATA4: GATA binding protein 4; GATA6: GATA binding protein 6; VEGFA: Vascular Endothelial Growth Factor A; TGF-β: transforming growth factor beta; TSP1: Thrombospondin-1; ITGA10: Integrin β1 subunit α10; ITGAM: Integrin β2 subunit M (synonym CD11b).

**Table 2:** Primers and probes used for real time qPCR.

Gene	NCBI Accession No	Location	Sequence (5′-3′)	PCR Product Length (bp)
*GAPDH*	XM_006933438.4	F: 57	F: GCCGTGGAATTTGCCGT	82
		R: 138	R: GCCATCAATGACCCCTTCAT	
		P: 77 [90]	P: CTCAACTACATGGTCTACATGTT-CCAGTATGATTCCA	
*IGF1R*	XM_023254968	F: 975	F: GCGTCTCCGAAATTTACCGC	111
		R: 1085	R: CGTCACTTTCACAGGAGGCT	
		P: 1015	P: TAAGGGGCGCCAGAGCAAAG	
*IRS1*	XM_003991219.4	F: 4179	F: GCGAGGATTTAAGCGCCTATG	99
		R: 4277	R: CATTCAGGTCTTCATTCTGCTGT	
		P: 4207	P: CAGTTTCCAGAAGCAGCCAGA	
*MRAS*	XM_019810658.3	F: 409	F: CGGCTTCCTCATCGTCTACTC	104
		R: 512	R: TCGGAAATGACTCTCTGTCCT	
		P: 470	P: CACCAGCTCATCCTGCGCGT	
*PI3K*	XM_019840086.3	F: 3818	F: AGACGACTTTGTGACCTGCG	111
		R: 3928	R: CATGCCAATAGCAAAACCAATTTCT	
		P: 3865	P: TGAGCCAGTAGGCAACCGTGA	
*B-RAF*	XM_019825910.3	F: 1456	F: TTGGATCCGGGTCATTTGGG	147
		R: 1602	R: TCACATGCCGAGTTTTCCTGA	
		P: 1530	P: TGACAGCACCCACACCTCAGC	
*MFN2*	XM_019836178.3	F: 1008	R: AAGAAGATGCGGTCTCCAGC	104
		R: 1111	P: CTGGGCGTGGTGGACCGAGGC	
		P: 1068	R: AAGAAGATGCGGTCTCCAGC	
*GATA4*	XM_011281564.4	F: 1771	F: CATGGCCCAGACGTTCTCA	119
		R: 1890	R: TGTGGTGACTGGCTGACAGAAG	
		P:1835	P: TCGGCCCTGAAGCTCTCCCC	
*GATA6*	XM_023241809.2	F: 1629	F: AGTCAAAAGCTTGCTCTGGTAG	119
		R: 1747	R: TGAGGATGTCGGTTGTGTTGT	
		P: 1661	P: TCTGTTCCTATGACTCCAACTTC	
*MEF2c*	XM_019836585.2	F: 501	F: ATCTTCAACAGCACCAACAAGCT	155
		R: 632	R: TTCTCAACGTCTCCACGATGTC	
		P: 567	P: TACACGGAGTACAACGAGCCGCAC	
*VEGFA*	XM_045057004.1	F: 1047	F: CTTCAAGCCATCCTGCGTG	113
		R: 1159	R: TGATCCGCATAATCTGCATGG	
		P: 1102	P: CGAGGGCCTGGAGTGTGTGC	
*TSP1*	XM_011283077.4	F: 2124	F: CGCCAACAAACAGGTATGCAA	118
		R: 2241	R: CTCACAGCGGTACATGGGG	
		P: 2147	P: CCCGCAACCCCTGCACAGAT	
*ITGAM*	XM_006942117.4	F: 938	F: TCGCTATGTCATTGGGGTGG	102
		R: 1039	R: ACCCGGAACACATAATCACGA	
		P: 993	P: CTTAATACCATTGCATCAAAGCCT	
*ITGA10*	XM_019837750.3	F: 3546	F: AGCAACACTCGGTGTCAAGT	122
		R: 3667	R: GACTTGAATTTGGCCCTCCG	
		P: 3575	P: CCATCCTTGGGCGGCTGGCAA	

Abbreviations: F: Forward primer; R: Reverse primer; P: Probe; bp: base pairs; GAPDH: glyceraldehyde 3-phosphate dehydrogenase; IGF1R: Insulin growth factor receptor 1; IRS1: Insulin receptor substrate 1; MRAS: Ras-related protein M-RAS isoform; PI3K: Phosphoinositide 3-Kinase; B-RAF: serine-threonine-protein kinase B-raf; MFN2: Mitofusin 2; GATA4: GATA binding protein 4; GATA6: GATA binding protein 6; MEF2c: Myocyte enhancer factor 2c.

## Data Availability

The original contributions presented in this study are included in the article and Supplementary Materials. Further inquiries can be directed to the corresponding authors.

## Supplementary Materials

The following supporting information can be downloaded at https://www.mdpi.com/article/10.3390/ijms26136497/s1.

## Abbreviations

The following abbreviations are used in this manuscript:

ANOVA	Analysis of Variance
B-RAF	Serine-threonine-protein kinase B-raf
cDNA	complementary deoxyribonucleic acid
DNA	Deoxyribonucleic acid
ERK1/2	Extracellular signaling-regulated kinase 1/2
GAPDH	Glyceraldehyde 3-phosphate dehydrogenase
GATA4	GATA binding protein 4
GATA6	GATA binding protein 6
IGF1R	Insulin-like growth factor 1 receptor
INSR	Insulin receptor
IRS1	Insulin receptor substrate 1
ITGA10	Integrin β1 subunit α10
ITGAM	Integrin β2 subunit M (synonym CD11b)
MAPK	Mitogen-activated protein kinase
MEF2C	Myocyte enhancer factor 2C
MFN2	Mitofusin 2
MMP	Matrix metalloproteinase
MRAS	RAS-related protein M-Ras isoform
NCBI BLAST	National Library of Medicine Basic Local Alignment Search Tool
PCR	Polymerase chain reaction
PI3K	Phosphatidylinositol 3-kinase
RNA	Ribonucleic acid
RT	Reverse transcription
RT-qPCR	Real-time quantitative polymerase chain reaction
TGF-β	Transforming growth factor beta
TIMP	Tissue inhibitor of metalloproteinase
TSP1	Thrombospondin-1
VEGFA	Vascular Endothelial Growth Factor A
