# Supplementary material for: Selected Pathway Analyses to Gain Mechanistic Insights into the Pathogenesis of Feline Hypertrophic Cardiomyopathy
Source: Int J Mol Sci. 2025 Jul 5;26(13):6497. doi: 10.3390/ijms26136497 (PMC12249748; doi:10.3390/ijms26136497)
Supplement: Supplementary file 1 [file ijms-26-06497-s001.zip › ijms-3697424-supplementary.pdf]

Supplemental Table S1. Study populations with information on clinical signs and additional findings (cats with HCM) or main pathological finding (control cats).

A) Cats with HCM. Breed, sex, age, clinical signs and additional pathological changes.

Cat No	Breed	Sex	Age (y)	Clinical signs	Additional pathological changes
1.1	Bengal	MN	9	Dyspnea	SAM
1.2	Sphynx	M	1	Dyspnea	-
1.3	Maine Coon	MN	6	Dyspnea	-
1.4*	ESH	MN	15	Reduced condition	SAM
1.5*	Chartreux	MN	15	Dyspnea, paraplegia	ATE
1.6	ESH	M	12	Paraplegia	-
1.7	ESH	F	13	Paraplegia	ATE
1.8	Scottish Fold Shorthair	M	9	Dyspnea, paraplegia	ATE
1.9	ESH	FN	10	Dyspnea	SAM
1.10	ESH	FN	12	Dyspnea	-
1.11	ESH	FN	14	Dyspnea	MV stenosis
1.12	ESH	MN	6	Dyspnea	-
1.13	ESH	MN	5	Dyspnea	-
1.14	BSH	MN	3	Dyspnea	-
1.15	Scottish Fold Shorthair	MN	4	Dyspnea	-

Abbreviations: ESH: European Shorthair; BSH: British Shorthair; F: female; FN: female neutered; M: male; MN: male neutered; SAM: systolic anterior motion; ATE: arterial thromboembolism; MV: mitral valve. * = full post mortem examination performed

B) Control cats. The list only comprises the older adult control animals. For these the exact age was not known, therefore only breed and sex are provided, together with the main pathological findings. The younger control cats (cats 3.1 to 3.16) are not listed as they were all of the same age (1.5 years) and clinically healthy; they did not exhibit any pathological changes.

**Cat No**	**Breed**	**Sex**	**Main pathological finding**
2.1	DSH	F	Intestinal invagination
2.2	DSH	M	Urinary bladder rupture
2.3	DSH	M	Trauma
2.4	DSH	M	Trauma
2.5	DSH	M	Trauma
2.6	DSH	F	Trauma
2.7	DSH	M	Trauma
2.8	DSH	M	Trauma
2.9	DSH	F	Trauma
2.10	DSH	M	Trauma
2.11	DSH	F	Trauma
2.12	DSH	M	Trauma
2.13	DSH	M	Trauma
2.14	DSH	M	Trauma

Abbreviations: DSH: Domestic Shorthair; F: female; M: male

Supplemental Table S2. Results of the statistical analyses performed in R for all markers in cats with HCM (Group 1), and both control cat groups (young controls: Group 2; older adult controls: Group 3).

	Markers												
	**IGF1R**	**IRS1**	**MRAS**	**PI3K**	**B-RAF**	**MFN2**	**GATA4**	**GATA6**	**MEF2C**	**VEGFA**	**TSP1**	**ITGA10**	**ITGAM**
Shapiro-Wilk													
Raw (p-value)													
1	5.42E-04	1.77E-03	2.16E-04	6.32E-04	2.69E-04	3.69E-04	1.02E-05	1.51E-04	3.87E-04	7.44E-05	1.78E-03	1.67E-04	6.47E-03
2	3.02E-01	5.85E-07	4.44E-03	4.74E-01	6.47E-02	1.03E-03	2.49E-04	3.43E-01	1.58E-03	4.72E-04	9.72E-03	4.80E-01	8.53E-03
3	8.72E-05	2.50E-05	7.42E-01	9.63E-05	2.37E-04	7.50E-04	1.55E-02	1.44E-05	1.49E-04	4.71E-05	1.07E-06	1.02E-02	5.63E-05
Log$_{10}$ (p-value)													
1	8.40E-01	1.03E-01	2.61E-01	5.03E-02	7.94E-02	9.15E-02	9.30E-01	6.25E-02	1.35E-01	9.67E-01	5.52E-01	5.63E-01	4.12E-01
2	2.09E-01	7.74E-05	7.33E-02	9.99E-01	4.71E-01	1.03E-01	2.57E-01	2.45E-01	4.52E-01	1.23E-01	2.94E-01	1.05E-02	9.58E-01
3	6.03E-01	5.83E-02	2.44E-01	5.36E-01	7.44E-01	8.04E-01	9.00E-01	7.51E-02	6.37E-01	2.53E-01	2.30E-02	8.35E-01	3.20E-01
ANOVA 1w													
Group°	1.91E-07 ***	5.38E-07 ***	1.73E-09 ***	4.46E-15 ***	9.43E-07 ***	1.81E-04 ***	3.00E+09 ***	1.18E-14 ***	2.02E-13 ***	4.76E-08 ***	2.65E-08 ***	1.77E-06 ***	5.20E-04 ***
Tukey													
1-3	2.09E-04 ***	1.76E-01	1.14E-04 ***	3.42E-03 **	1.67E-01	4.27E-03 **	9.28E-01	1.22E-02 *	7.44E-02	6.71E-02	8.10E-06 ***	1.01E-06 ***	1.68E-03 **
2-3	1.23E-07 ***	5.78E-07 ***	8.00E-10 ***	1.07E-12 ***	9.39E-07 ***	1.83E-04 ***	2.29E-04 ***	1.09E-12 ***	1.69E-12 ***	4.23E-08 ***	3.21E-08 ***	6.61E-04 ***	9.64E-01

2-1	7.41E-02	1.81E-04 ***	2.20E-03 **	1.00E-10 ***	3.15E-04 ***	5.68E-01	5.76E-04 ***	1.00E-10 ***	4.00E-10 ***	6.78E-05 ***	2.93E-01	6.07E-02	2.03E-03 **
ANOVA 2w													
Group°	1.69E-08 ***	1.34E-07 ***	4.09E-11 **	7.94E-16 ***	3.59E-07 ***	9.73E-05 ***	2.58E-05 ***	8.62E-16 ***	5.07E-14 ***	4.75E-08 ***	2.81E-08 ***	4.24E-07 ***	1.10E-04 ***
Sex°	1.35E-01	6.88E-01	2.86E-01	2.99E-01	7.92E-01	1.52E-01	8.94E-01	7.57E-01	2.56E-01	8.88E-01	4.07E-01	6.49E-01	2.05E-01
Group*Sex°	4.40E-03 **	1.07E-02 *	5.97E-04 ***	1.01E-02 *	2.19E-02 *	7.79E-02	1.44E-02 *	2.39E-03 **	1.90E-02 *	1.20E-01	1.72E-01	1.03E-02 *	4.00E-03 **

Tukey													
1:F-3:F	9.96E-01	1.00E+00	9.68E-01	9.98E-01	9.89E-01	1.00E+00	3.00E-01	1.00E+00	9.91E-01	9.73E-01	4.90E-01	3.38E-01	9.50E-01
2:F-3:F	1.38E-04 ***	2.06E-04 ***	3.75E-07 ***	1.10E-09 ***	1.42E-03 **	5.12E-02	1.98E-01	1.90E-09 ***	1.89E-07 ***	6.94E-05 ***	6.54E-04 ***	2.30E-02 *	6.40E-01
3:M-3:F	1.00E+00	1.00E+00	9.97E-01	9.98E-01	1.00E+00	1.00E+00	7.47E-01	1.00E+00	1.00E+00	9.89E-01	1.00E+00	1.00E+00	9.08E-01
2:F-1:F	8.03E-04 ***	6.49E-05 ***	6.69E-06 ***	4.30E-09 **	1.62E-04 ***	1.14E-01	3.20E-04 ***	9.00E-10 ***	2.27E-08 ***	1.01E-03 **	1.43E-01	8.67E-01	9.94E-01
1:M-1:F	7.67E-03 **	1.45E-03 **	5.11E-03 **	4.19E-02 *	1.53E-01	8.92E-02	1.51E-01	3.53E-02 *	4.14E-02 *	7.34E-01	3.41E-01	9.20E-02	2.54E-02 *
2:M-2:F	9.22E-01	1.82E-01	3.51E-01	7.51E-01	6.60E-01	1.00E+00	5.70E-03 **	3.13E-01	9.63E-01	6.76E-01	9.97E-01	5.02E-01	5.75E-01
1:M-3:M	3.82E-05	1.56E-01	1.41E-05	2.21E-03	1.10E-01	3.97E-03	5.07E-01	3.04E-03	3.27E-02	2.40E-01	5.09E-05	1.66E-06	1.66E-03

	***		***	**		**		**	*		***	***	**
2:M-3:M	8.06E-05 ***	3.80E-03 **	3.45E-06 ***	2.00E-10 ***	1.58E-03 **	1.58E-02 *	1.45E-02 *	5.00E-10 ***	4.10E-09 ***	7.93E-04 ***	6.88E-05 ***	1.40E-01	9.11E-01
2:M-1:M	1.00E+00	5.26E-01	9.39E-01	1.76E-05 ***	4.36E-01	1.00E+00	4.12E-01	3.36E-05 ***	2.69E-05 ***	1.54E-01	1.00E+00	5.70E-03 **	1.04E-04 ***

Abbreviations: F: female; M: male; °: categorical variables used for the ANOVA test (1w: one-way; 2w: two-way), Group (1, 2, 3), Sex (F, M); Asterisks indicate the significance level: p-value/adjusted p-value ≤0.05 (*); p-value/adjusted p-value ≤0.01 (**); p-value/adjusted p-value ≤0.001 (***)
